# Essays on Infant Therapeutics

**Published:** 1852-01

**Authors:** 


ARTICLE XIV.

*Essays on Infant Therapeutics: To which are added observations on Ergot; History of the use of Mercury in Inflammatory Complaints; Together with the Statistics of the deaths from Poisoning in New York in the years* 1841--2--3. By JOHN B. BECK, M. D., Professor of Mat. Med. College of Physicians and Surgeons, N. Y., &c., &c. Second edition, enlarged and revised. Pages 168. New York: William E. Dean, Publisher, No. 2 Ann Street, 1852. (By mail.)

Several of the Essays making up this volume were published in our Journal some years ago, and subsequently a favorable notice of the appearance of the first edition of the work. We now express our pleasure in chronicling the appearance of a new edition.

This little work is one of the best productions of its distinguished author. It treats of several of the most active and important therapeutical agents in their application to children.

